# Apoptosis is induced in both drug-sensitive and multidrug-resistant hepatoma cells by somatostatin analogue TT-232

**DOI:** 10.1038/sj.bjc.6690486

**Published:** 1999-06

**Authors:** C-C Diaconu, M Szathmári, G Kéri, A Venetianer

**Affiliations:** Institute of Genetics, Biological Research Center, Hungarian Academy of Sciences, PO Box 521, Szeged, H-6701, Hungary; Department of Medical chemistry, Molecular Biology and Pathobiochemistry, Joint Research Organization of the Hungarian Academy of Sciences and Semmelweis University Medical School, Budapest, Hungary

**Keywords:** somatostatin analogue, apoptosis, hepatoma, multidrug-resistant

## Abstract

Clinical resistance to chemotherapeutic drugs is an important problem in the treatment of cancer; the circumvention of resistance has become one of the basic goals of cancer therapy. The most frequent form of primary liver cancer is hepatocellular carcinoma, which is essentially refractory to chemotherapy. We earlier showed that TT-232, a new somatostatin analogue developed in our laboratory, exerted a strong antiproliferative effect both in vitro and in vivo, but no growth hormone release inhibitory or antisecretory activity. Here we report that TT-232 has a pronounced antiproliferative effect on differentiated and dedifferentiated, drug-sensitive and multidrug-resistant hepatocellular carcinoma cell lines. TT-232 induces apoptosis at comparable levels in all these hepatoma variants demonstrating that the multidrug resistance of hepatomas does not correlate with a reduced susceptibility to apoptosis induction. These results clearly reveal that the machinery involved in apoptosis is functional in both drug-sensitive and resistant hepatoma variants and can be activated by the somatostatin analogue TT-232. © 1999 Cancer Research Campaign


*British Journal of Cancer* (1999) **80**(8), 1197–1203
© 1999 Cancer Research Campaign
Article no. bjoc.1998.0486

### Apoptosis is induced in both drug-sensitive and multidrug-resistant hepatoma cells by somatostatin analogue TT-232

**C-C Diaconu[1], M Szathmári[1], G Kéri[2] and A Venetianer[1]**

[1]Institute of Genetics, Biological Research Center, Hungarian Academy of Sciences, H-6701 Szeged, PO Box 521, Hungary; Joint Research Organization of the Hungarian Academy of Sciences and Semmelweis University Medical School, Department of Medical Chemistry, Molecular Biology and Pathobiochemistry, Budapest, Hungary

**Summary** Clinical resistance to chemotherapeutic drugs is an important problem in the treatment of cancer; the circumvention of resistance has become one of the basic goals of cancer therapy. The most frequent form of primary liver cancer is hepatocellular carcinoma, which is essentially refractory to chemotherapy. We earlier showed that TT-232, a new somatostatin analogue developed in our laboratory, exerted a strong antiproliferative effect both in vitro and in vivo, but no growth hormone release inhibitory or antisecretory activity. Here we report that TT-232 has a pronounced antiproliferative effect on differentiated and dedifferentiated, drug-sensitive and multidrug-resistant hepatocellular carcinoma cell lines. TT-232 induces apoptosis at comparable levels in all these hepatoma variants demonstrating that the multidrug resistance of hepatomas does not correlate with a reduced susceptibility to apoptosis induction. These results clearly reveal that the machinery involved in apoptosis is functional in both drug-sensitive and resistant hepatoma variants and can be activated by the somatostatin analogue TT-232.

**Keywords:** somatostatin analogue; apoptosis; hepatoma; multidrug-resistant

Clinical resistance to chemotherapeutic drugs is an important problem in the treatment of cancer. Tumour cells can acquire resistance to a wide variety of unrelated cytotoxic drugs when they are exposed to a chemotherapeutic agent. This phenomenon is called multidrug resistance (MDR). The molecular basis of a major form of MDR is the overexpression of P-glycoprotein (P-gp), which functions as an adenosine 5′-triphosphate (ATP)-dependent efflux pump for several structurally and functionally unrelated cytotoxic drugs (Endicott and Ling, 1989; Gottesmann, 1993). The cirumvention of resistance to chemotherapy has become one of the primary goals of modern approaches to cancer therapy.

Several chemotherapeutic drugs kill drug-sensitive cells by apoptosis (Dyson et al, 1986; Muller et al, 1997). Physiological or apoptotic cell death is an active, genetically programmed suicidal process that consists of a cascade of well-regulated events with the participation of specific genes (Wyllie et al, 1980; Sen and D'Incalci, 1992). The three main stages of programmed cell death are signalization, control and execution (Golstein, 1997). Disturbance of any of these stages may result in the failure of the apoptotic programme. Resistance to apoptosis has emerged as an important category of treatment resistance (Fisher, 1994). Recent research efforts have concentrated on the possibility of the manipulation of apoptotic cell death in order to intervene therapeutically in cancer, where failure or suppression of apoptosis is likely to contribute both to the initial development of cancer and to the appearance of tumour cells resistant to chemotherapy.

*Received 6 March 1998*
*Revised 29 July 1998*
*Accepted 8 September 1998*

*Correspondence to:* A Venetianer

Somatostatin is a tetradecapeptide with diverse biological effects on cellular functions, including the inhibition of secretory and proliferative processes (Brazeau, 1973; Schally, 1988). These effects are mediated by specific receptors that belong to the guanine nucleotide binding protein-linked receptor family (Patel et al, 1995).

In a search for tumour-selective somatostatin analogues, we earlier found that one of our analogues, TT-232 (D-Phe-Cys-D-Trp-Lys-Cys-Thr-$NH_2$), exerted a strong antiproliferative effect, but had practically no growth hormone release inhibitory or antisecretory activity (Kéri et al, 1993*a*, 1993*b*). This compound has a substantially different structure from the growth hormone active analogues (Jaspers et al, 1994). We have demonstrated that TT-232 exerts a strong in vitro and in vivo anti-tumour activity (Kéri et al, 1996).

The most frequent form of primary liver cancer is hepatocellular carcinoma, which causes more than 300 000 deaths per year (Ozturk, 1996). Hepatocellular carcinoma cells are essentially refractory to chemotherapy. The aim of our recent work was to examine whether TT-232 exerts antiproliferative effects on hepatoma cells and whether apoptosis can be triggered in hepatomas. An important part of this work was to test the antiproliferative effect of TT-232 on multidrug-resistant hepatoma cells. We earlier isolated stable, heat-resistant rat hepatoma variants by using repeated heating cycles at 45°C (Venetianer et al, 1994). These heat-resistant variants became moderately multidrug-resistant, they expressed a slightly elevated level of P-gp and an increased basal level of several heat-shock proteins (Venetianer et al, 1994; Pirity et al, 1996). Highly multidrug-resistant hepatoma variants were also isolated with increasing concentrations of colchicine, these cells strongly overexpressed functional P-gp. Our closely related hepatoma variants representing different degrees of MDR offered a valuable tool to test whether the new somatostatin analogue TT-232 can inhibit cell proliferation or induce apoptosis in the different hepatoma cells.

### MATERIALS AND METHODS

### Cells lines, media, culture conditions

The culture conditions have been described elsewhere (Venetianer et al, 1980). Briefly, the cells were grown in F12 (GibcoBRL) medium supplemented with 5% fetal calf serum (BIO MEDIA) in a humidified atmosphere of 8% carbon dioxide in air.

The glucocorticoid-resistant clone 2 cells (Venetianer et al, 1980) were isolated from the glucocorticoid-sensitive, differentiated rat hepatoma Faza 967 clone (Deschatrette and Weiss, 1974). Clone 2 cells were grown for several years in the presence of 2 μM dexamethasone, and lost the ability to express all liver-specific functions examined, i.e. they became dedifferentiated (Venetianer et al, 1983).

Stable heat-resistant cells of clone 2 were isolated by ten repeated cycles of heat exposure at 45°C for 80 min. A subclone derived from them and used in the present studies is referred to as clone 2(10×80)T1 (Venetianer et al, 1994). The heat-resistant derivatives of clone 2 cells were moderately multidrug-resistant. The relative resistance of clone 2(10×80)T1, determined with Trypan blue exclusion test after treatment for 72 h with various concentrations of drugs was the following: 5.2 for vinblastine, 9.4 for doxorubicin, 11.4 for puromycin, 7.7 for actinomycin D and 2.4 for colchicine (Pirity et al, 1996).

The highly multidrug-resistant variants were isolated from clone 2 cells (clone $2_{c1000}$) and from the moderately multidrug-resistant clone 2(10×80)T1 (clone 2(10×80)$T1_{c1000}$) by using step-wise selection with colchicine, and thereafter grown continuously in the presence of 1000 ng $ml^{-1}$ colchicine. The relative resistance of clone $2_{c1000}$ and clone 2(10×80)$T1_{c1000}$ was found to be: 34 and 5.7 for vinblastine, 8.8 and 14.6 for doxorubicin, 65.9 and 65.6 for puromycin, 21.3 and 36.3 for actinomycin D and 37.4 and 40.7 for colchicine respectively (Pirity et al, 1996). The MDR of the variants correlated with the overexpression of the functional P-gp (Pirity et al, 1996). The highly multidrug-resistant variants were grown in the absence of colchicine for a week before performing viability tests or other assays.

### Cell viability tests

Equal numbers of cells were plated into 35-mm Petri dishes. One day later, the cells were treated with different concentrations of TT-232 and the numbers of viable cells were determined by the Trypan blue exclusion test 24, 48 and 72 h later. For evaluation of the results, it should be considered that in cultured cells the cell membrane does not become permeable to vital dyes in the first and second stages of apoptosis, but only in the third stage; as the membranes rupture, the cells become permeable (Arends and Willie, 1991).

#### *XTT assay*

Equal numbers of cells were plated into 96-well tissue culture plates. One day later, the cells were treated with different concentrations of TT-232. Cell viability was determined in triplicate wells for each drug concentration using the XTT/PMS viability dye assay (Scudiero et al, 1988; Roehm et al, 1991) 24, 48 and 72 h later. Cell viability was expressed as the per cent of absorbance of treated cells relative to the untreated control wells, measured at 450 nm.

The concentrations of drugs which reduced the viability of the treated cells by 50% of that of controls ($IC_{50}$ values) were determined using the Trypan blue exclusion test and the XTT assay.

### Colony-forming ability after drug exposure

Aliquots of about 1000 dispersed cells were seeded into Petri dishes in triplicates and treated with drugs 16 h later (control cells were not treated); culture media were renewed weekly. Clone 2(10×80)T1 and clone 2(10×80)$T1_{c1000}$ cells were grown for 11 days, clone 2 and clone $2_{c1000}$ for 17 days, thereafter the plates were fixed, stained and counted, as described previously (Venetianer et al, 1978; Pirity et al, 1992).

### Assay for induction of apoptosis

A total of $1 \times 10^5$ (for propidium iodide) or $5 \times 10^4$ (for cytospin) cells were seeded in 35-mm Petri dishes with or without coverslips and treated with different concentrations of TT-232 1 day later. After exposure, cells seeded on coverslips were fixed, washed and subsequently stained with propidium iodide as described earlier (Bonfoco et al, 1995). Coverslips were examined either with a Zeiss, Axiovert 135 M confocal laser scanning microscope or with a Leitz fluorescence microscope. Nuclei of untreated cells revealed a typical chromatin morphology with distinct organization, whereas apoptotic nuclei were highly fluorescent, condensed and displayed polarized chromatin aggregates. For quantitative evaluation of apoptotic cells, 20 fields (at least 200 cells) were counted in each preparation. In parallel, the percentage of cells with morphological characteristics of apoptosis and necrosis was determined on a May–Grünwald–Giemsa-stained cytospin preparation (Shandon Elliot cytocentrifuge) by counting at least 400 cells.

### Confocal microscopy

Confocal microscopic studies were performed with a Zeiss, Axiovert 135 M, LSM 410 Laser Scan Confocal Microscope and a 100× (N.A. = 1.5, oil) objective. The *z*-axis position was fixed so that the optical section clearly transected the nuclei. Fluorescence was excited by using an argon ion laser at 488 nm (15 mW). Nuclei area measurements for at least 20 randomly selected fields of propidium iodide-stained TT-232-treated and control cells were performed using the LSM software, which is based on the Microsoft Windows graphical user interface.

### DNA ladder assay

Clone 2 and clone 2(10×80)$T1_{c1000}$ cells were treated with 30 μg $ml^{-1}$ TT-232 for 21 h; control cells were not treated. As an internal control for ladder formation, drug-sensitive clone 2 cells were treated with 60 ng $ml^{-1}$, highly drug-resistant clone 2(10×80)$T1_{c1000}$ cells with 600 ng $ml^{-1}$ actinomycin D for 21 h. Fragmented DNA was separated from intact DNA, extracted and loaded onto agarose gels as described earlier (Laurent-Crawford et al, 1991). *PstI*-digested λ DNA was used as molecular weight marker.

*© 1999 Cancer Research Campaign*

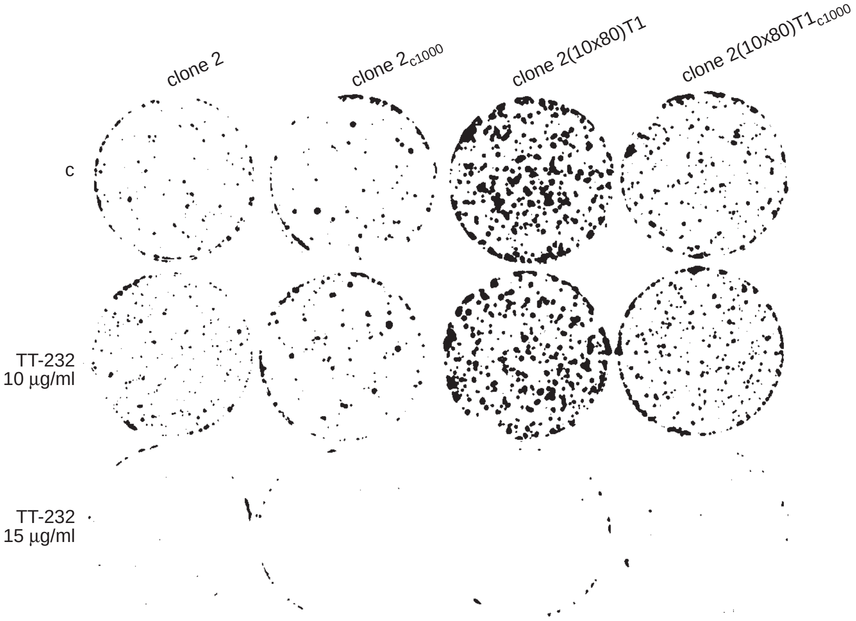

**Figure 1** Colony formation of hepatoma variants in the presence or absence (c) of different concentrations of TT-232

### Measurement of intracellular doxorubicin concentration

Hepatoma cells were exposed to 10 μM doxorubicin for 4 h, in the presence or absence of 10 µg $ml^{-1}$ TT-232. The intracellular concentration of doxorubicin was determined as described previously (Fardel et al, 1993).

### Peptides

TT-232 (D-Phe-Cys-Try-D-Trp-Lys-Cys-Thr-$NH_2$) was synthetized in our laboratory as described earlier (Kéri et al, 1993*a*) then, 2 mg $ml^{-1}$ TT-232 stock solution was prepared by dissolving TT-232 in 0.2 M acetic acid–sodium acetate buffer, pH 3.45 adjusted to 5% mannit (w/v) and stored at 4°C up to 1 month. Before use the TT-232 stock solution was diluted in culture medium. The vehicle was also added to control cultures. Chemicals were obtained from Sigma.

### RESULTS

### In vitro antiproliferative effect of TT-232 on hepatoma cell lines

XTT assay was used to compare the cytotoxicity of TT-232 on different variants. Cells were treated with different concentrations of TT-232 for 3 days. $IC_{50}$ values determined at least in three independent experiments were the following: 14 ± 1.8 µg $ml^{-1}$ for clone 2, 10 ± 0.7 µg $ml^{-1}$ for clone 2(10×80)T1, 12 ± 1.1 µg $ml^{-1}$ for clone 2(10×80)$T1_{c1000}$ and 12 ± 1.5 for the drug-sensitive, differentiated Faza 967. The effect of TT-232 on cell viability was also determined using the Trypan blue exclusion test following a 1-day treatment. The $IC_{50}$ were found to be between 45 and 54 µg $ml^{-1}$ (54 ± 3 µg $ml^{-1}$ for clone 2; 53 ± 4 µg $ml^{-1}$ for clone $2_{c1000}$; 45 ± 3 µg $ml^{-1}$ for clone 2(10×80)T1; 45 ± 4 µg $ml^{-1}$ for clone 2(10×80)$T1_{c1000}$; and 46 ± 3 µg $ml^{-1}$ for Faza 967 clone).

To examine the long-term effects of TT-232 on different hepatomas, the cells were treated continuously up to 17 days with the hormone analogue and the extents of colony formation were compared. While 10 µg $ml^{-1}$ TT-232 had no or only a slight effect on colony formation, 15 µg $ml^{-1}$ drastically inhibited colony formation in all hepatoma variants (Figure 1).

The results of the different assays clearly show that TT-232 inhibits the proliferation and viability of all examined hepatoma cells and the inhibition is dose- and time-dependent. These experiments reveal that the sensitivity of moderately- and highly multidrug-resistant variants to TT-232 was not reduced.

### TT-232 induces apoptosis in drug-sensitive and multidrug-resistant cells

In an attempt to characterize the mechanisms responsible for the cytotoxicity of TT-232 on hepatoma cells, we tested the possible apoptosis-inducing effect of the hormone analogue. Treatment of hepatoma cells with TT-232 induced the typical apoptotic morphology in both drug-sensitive (Figure 2A, B) and multidrug-resistant cells (Figure 2C, D). To determine the ratio of apoptotic and necrotic cells the variants were treated with TT-232 for 1, 2 and 3 days. The percentage of cells with morphological characteristics of apoptosis was determined on coverslips following propidium iodide staining and in May–Grünwald–Giemsa-stained cytospin preparations. Necrosis occurred at much lower rates than apoptosis in all TT-232-treated cells; 20 µg $ml^{-1}$ TT-232 induced approximately 20–40% apoptosis and 9–12% necrosis after treatment for 1 day, 30–60% apoptosis and 3–17% necrosis after treatment for 2 days, and 40–70% apoptosis and 9–28% necrosis after treatment for 3 days. Figure 2E, F depicts the percentages of apoptotic and necrotic cells in clone 2, clone $2_{c1000}$ and clone 2(10×80)$T1_{c1000}$ cells following treatment with TT-232 for 3 days. It is clear from the results that the MDR of hepatomas is not correlated with a reduced susceptibility to apoptosis induction by TT-232.

© 1999 Cancer Research Campaign

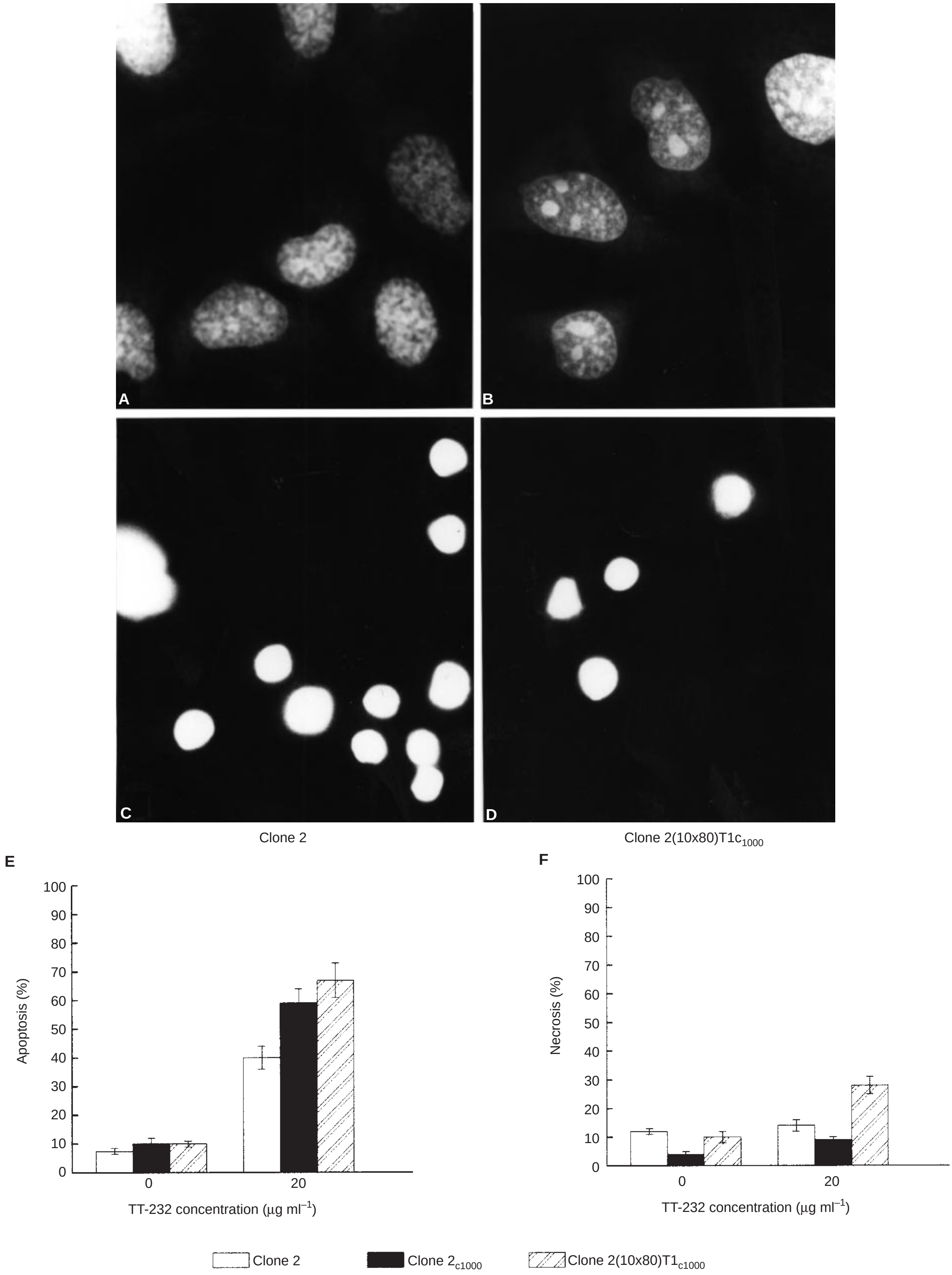

**Figure 2** TT-232 induces apoptosis in hepatoma variants. Morphological appearance of apoptotic cells. Drug-sensitive clone 2 (**C**) and multidrug-resistant clone 2(10×80)$T1_{c1000}$ (**D**) cells were treated with 40 µg $ml^{-1}$ TT-232 for 1 day and stained with propidium iodide. Control cells (**A, B**) were not treated. Apoptotic nuclei are highly fluorescent, condensed. Quantitation of apoptotic (**E**) and necrotic (**F**) cell death in hepatoma variants treated with 20 µg TT-232 for 72 h. Apoptotic cells are expressed as a percentage of the total propidium iodide-stained cells attached to coverslips. The percentage of necrotic cells was determined in May–Grunwald–Giemsa-stained cytospin preparations. Bars are the mean ± s.e.m. of the results of three experiments

 *© 1999 Cancer Research Campaign*

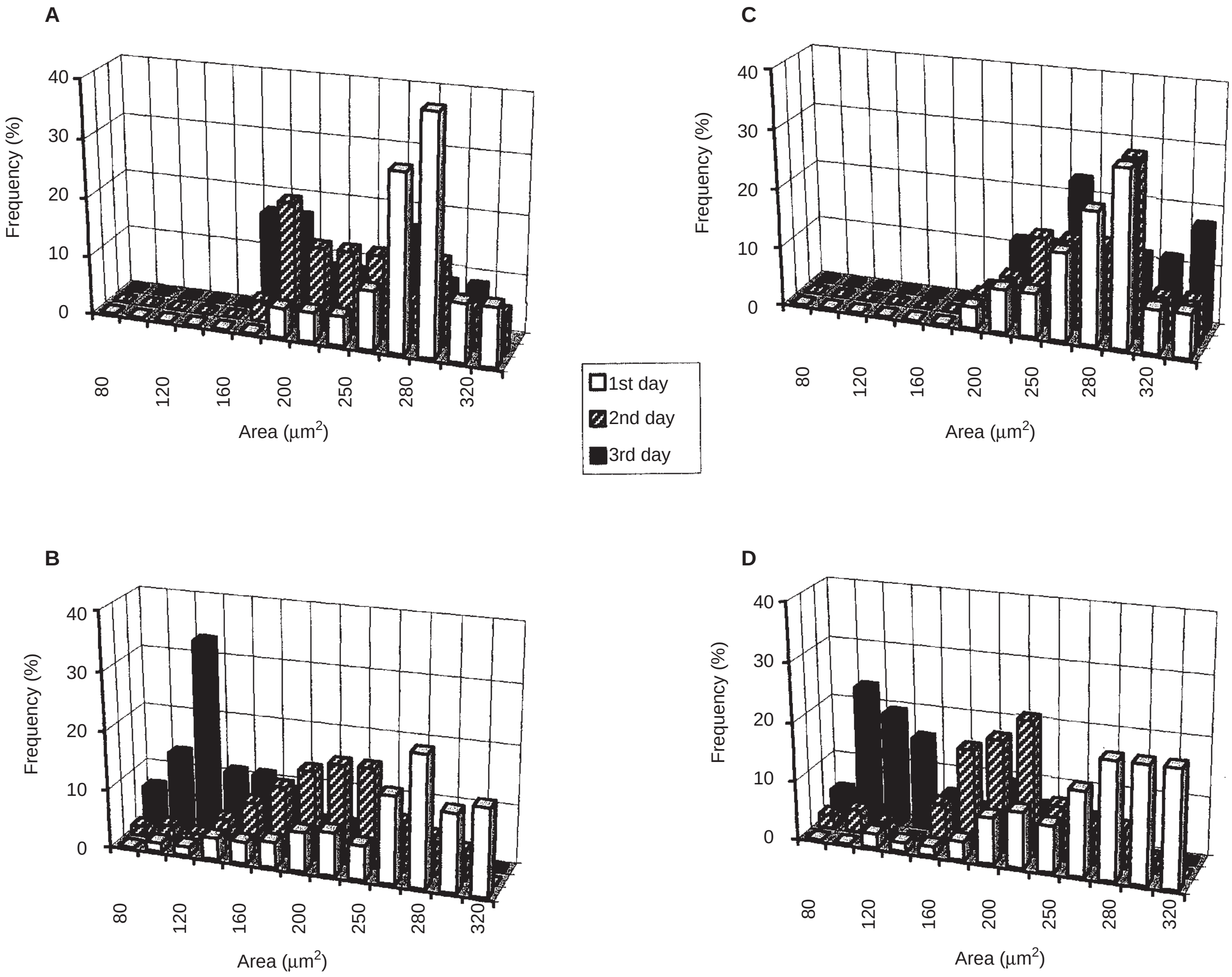

**Figure 3** Nuclear area distribution in clone 2 and clone $2_{c1000}$ cells following treatment with 20 μg ml$^{-1}$ TT-232 for 1, 2 and 3 days. Nuclei area measurements for at least 20 randomly selected fields were performed by using LSM software as described above. (**A, C**) Clone 2, clone $2_{c1000}$ control; (**B, D**) clone 2, clone $2_{c1000}$ treated

The apoptosis-inducing effect of TT-232 was also examined by measuring the areas of propidium iodide-stained nuclei with LSM software. During apoptosis, cell shrinkage and chromatin condensation occurred. Treatment of the hepatoma cells with TT-232 for 1, 2 and 3 days resulted in clear decreases in the areas of the nuclei. While the area of nuclei of the majority of control cells was around 300 μm$^2$, it was much lower, with highest frequencies around 140 μm$^2$, following treatment with 20 μg ml$^{-1}$ TT-232 for 3 days (Figure 3). A similar reduction in the size of the treated nuclei was seen for the highly drug-resistant hepatoma clone $2_{c1000}$ (Figure 3).

To learn whether treatment with increasing concentrations of TT-232 induces apoptosis in a dose-dependent manner, the drug-sensitive and resistant cells were treated with different concentrations of TT-232 for 1 day. The percentage of apoptotic cells increased in all hepatomas with increasing concentration of TT-232 and was between 60 and 75% at 60 μg ml$^{-1}$ TT-232, while the percentage of necrotic cells was significantly lower. These results reveal that the apoptosis-inducing effect of TT-232 is dose and time-dependent.

### Induction of DNA fragmentation in response to TT-232 treatment

One of the earliest steps in apoptosis appears to be DNA digestion. Apoptosis is usually associated with the activation of nucleases that degrade the chromosomal DNA first into large, and subsequently into very small, oligonucleosomal fragments.

Clone 2 and the multidrug-resistant cells were treated with 30 μg ml$^{-1}$ TT-232 for 21 h. For comparison, the cells were also treated with actinomycin D, which induces apoptosis and DNA ladder formation in these cells (unpublished results). A characteristic pattern of DNA fragments was observed in both drug-sensitive and resistant cells, which consisted of multimers of approximately 180 base pairs. A typical DNA fragmentation pattern is shown in Figure 4.

### TT-232 is not a substrate of P-glycoprotein

We reported earlier that the overexpression of P-gp in both moderately and highly multidrug-resistant hepatoma variants correlated

© 1999 Cancer Research Campaign

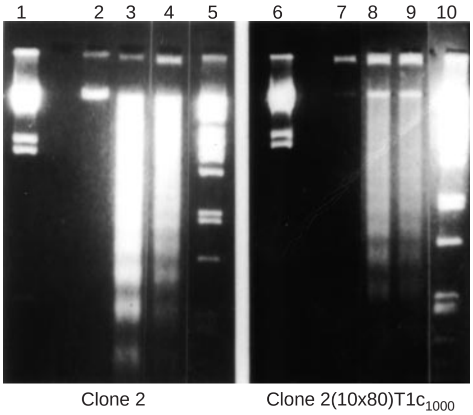

**Figure 4** DNA fragmentation in TT-232-treated hepatoma cells. Clone 2 cells were treated with 60 ng $ml^{-1}$ actinomycin D (lane 3), with 30 μg $ml^{-1}$ TT-232 (lane 4) for 21 h, control cells (lane 2) were untreated. Clone 2(10×80) $T1_{c1000}$ cells were treated with 600 ng $ml^{-1}$ actinomycin D (lane 8), with 30 μg $ml^{-1}$ TT-232 (lane 9) for 21 h, control cells were untreated (lane 7). Lanes 1, 6: γ *Hin*dIII; lanes 5,10: γ DNA/*Pst* 1. Fragmented DNA was separated from nucleoplasm, extracted and loaded onto agarose gels with a decreased accumulation of anticancer drugs, such as doxorubicin (Pirity et al, 1996). Addition of verapamil, a calcium channel blocker drug known to reverse MDR by inhibiting P-gp function, significantly increased the cellular doxorubicin accumulation. Addition of TT-232, however, had no effect on the doxorubicin retention. The intracellular concentration of doxorubicin in the absence and presence of 10 μg $ml^{-1}$ TT-232 was found to be: 38.5 ± 4 and 39.5 ± 4 μM $mg^{-1}$ protein in clone 2, and 1.2 ± 0.1 and 1.3 ± 0.1 μM $mg^{-1}$ in clone 2(10×80)$T1_{c1000}$ cells), indicating that TT-232 is not a substrate for P-gp.

### DISCUSSION

A structural analogue of somatostatin (TT-232) has been developed in our laboratory. TT-232 has been tested on various human tumour cell lines by the National Cancer Institute of NIH. It was found that 100 μM of TT-232 had a strong antiproliferative effect on the majority of cell lines. We recently reported that TT-232 induced apoptosis in different human tumour cell lines (Kéri et al, 1996). In vivo, TT-232 was effective on transplanted animal tumours (COLO26, B16 melanoma and S180 sarcoma) and on human tumour xenografts (Kéri et al, 1996). We found, for example, that 30% of S180 tumour-bearing mice treated twice daily intraperitonially with low dose (15 μg $kg^{-1}$) of TT-232 for 12 days became tumour-free, while continuous administration of the drug resulted in 60% tumour-free animals (Kéri et al, 1997). Our data suggest that TT-232 is a promising anti-tumour agent.

Primary cancer of the liver is the fourth most common cause of death from cancer (Ozturk, 1996). Hepatocellular carcinomas generally do not respond well to chemotherapy. Numerous mechanisms of drug resistance are well established for tumour cells in vitro (Fisher, 1994). The main goal of cancer chemotherapy is to kill tumour cells, but not their normal counterparts, with specific antineoplastic drugs. One promising way for doing this is to induce the genetically encoded death programme in cancer cells, including the multidrug-resistant tumour cells.

In this report, we have demonstrated for the first time that TT-232 has pronounced antiproliferative effects on hepatocellular carcinoma cell lines, including differentiated and dedifferentiated, drug-sensitive and multidrug-resistant hepatomas. Long-term treatment of the hepatomas with 15 μ $ml^{-1}$ TT-232 drastically inhibited colony formation in all examined hepatoma variants. The $IC_{50}$ values determined by the Trypan blue exclusion test were in the range 40–50 μM, indicating comparable sensitivities of the variants. The $IC_{50}$ values for breast, colon and prostate lines were in the range 20–40 μM (Kéri et al, 1996).

The most important finding in our experiments was that TT-232 induces apoptosis in all examined hepatoma variants. Treatments of hepatoma cells with TT-232 induced a typical apoptotic morphology, with a clear decrease in the areas of the nuclei and a characteristic pattern of DNA fragmentation. The major form of cell death occurring in the different hepatoma variants was apoptosis. The percentages of apoptotic cells were comparable following treatment with TT-232 in the drug-sensitive and highly multidrug-resistant hepatomas. Multidrug-resistant hepatoma variants overexpressing functional P-gp were found not to be less sensitive to TT-232 than the P-gp non-expressing, drug-sensitive cells. These observations can be explained by our observations showing that TT-232 is not a substrate of P-gp. These results clearly reveal that the machinery involved in apoptosis is functional both in drug-sensitive and in drug-resistant hepatoma variants, and it can be activated by the somatostatin analogue TT-232.

Our earlier results showed that the basal expression of several heat-shock proteins was increased in the heat-resistant, moderately multidrug-resistant hepatoma variants and in the highly multidrug-resistant derivatives of them (Venetianer et al, 1994; Pirity et al, 1996). Heat-shock proteins play an important role in protecting cells from the harmful effects of stress (Morimoto et al, 1990) and could also protect cells from apoptotic cell death (Mosser et al, 1992; Mehlen et al, 1996). Our data presented in this paper indicate that the observed increased basal expression of Hsp27 and Hsp70 did not prevent the initiation of apoptotic pathway in hepatoma cells treated with TT-232.

In recent years, considerable effort has been directed towards the development of small molecular tyrosine kinase inhibitors as potential anti-tumour drugs (Levitzki and Gazit, 1995). It was demonstrated that somatostatin and other pepide hormones and analogues, which inhibit tyrosine kinase activity, can also induce apoptosis (Kéri et al, 1996). Although we could not measure the inhibitory effect of TT-232 on the tyrosine kinase activity of our hepatoma cells, due to the low basal activity of the enzyme, we observed that the apoptosis-inducing effect of a recently described, very potent tyrosine kinase inhibitor, which is a quinazoline derivative (PD153035; Fry et al, 1994), on multidrug-resistant hepatoma cells is comparable to that of TT-232 (unpublished results).

Our recent finding showing that TT-232 can kill not only hepatocellular carcinoma cells, but also the multidrug-resistant variants by apoptosis together with our earlier data demonstrating strong in vivo anti-tumour activity at relatively low doses, and the results of toxicity studies suggest that TT-232 is a promising anti-tumour agent.

### ACKNOWLEDGEMENTS

Recent research has been supported by grants from Dr János Bástyai Holczer Fund, the National Scientific Research Fund (OTKA T16060 and T025008) and Health Research Council 129/1996. We thank Mária Tóth, András Borka and Csilla Soltész for assistance in preparation of the manuscript.

 © 1999 Cancer Research Campaign

### REFERENCES

Arends MJ and Wyllie AH (1991) Apoptosis: mechanisms and roles in pathology. *Int Rev Exp Pathol* **32**: 223–254

Bonfoco E, Ceccatelli S, Manzo L and Nicotera P (1995) Colchicine induces apoptosis in cerebellar granule cells. *Exp Cell Res* **218**: 189–200

Brazeau P, Vale W, Burgus R, Ling N, Butcher M, Rivier J and Guillemin R (1973) Hypothalamic polypeptide that inhibits the secretion of immunoreactive pituitary growth hormone. *Science* **179**: 77–79

Deschatrette J and Weiss MC (1974) Characterization of differentiated and dedifferentiated clones from a rat hepatoma. *Biochimie* **56**: 1603–1611

Dyson JED, Simmons DM, Daniel J, McLaughlin JM, Quirke P and Bird CC (1986) Kinetic and physiological studies of cell death induced by chemotherapeutic agents and hyperthermia. *Cell Tissue Kinetics* **19**: 311–324

Endicott JA and Ling V (1989) The biochemistry of P-glycoprotein-mediated multidrug resistance. *Annu Rev Biochem* **58**: 137–171

Fardel O, Lecereur V and Guillouzo A (1993) Regulation by dexamethasone of P-glycoprotein expression in cultured rat hepatocytes. *FEBS Lett* **27**: 189–193

Fisher DE (1994) Apoptosis in cancer therapy: crossing the threshold. *Cell* **78**: 539–542

Fry DW, Kraker AJ, Mcmichael A, Ambroso LA, Nelson JM, Leopold WR, Connors RW and Bridges AJ (1994) A specific inhibitor of the epidermal growth factor receptor tyrosin kinase. *Science* **265**: 1093–1095

Golstein P (1997) Controlling cell death. *Science* **275**: 1081–1082

Gottesman MM (1993) How cancer cells evade chemotherapy. *Cancer Res* **53**: 747–754

Jaspers H, Horváth A, Mezö I, Kéri G and Van Binst G (1994) Conformational study of somatostatin analogues with antitumor and/or GH inhibitory activity. *Int. J. Pept. Protein Res* **43**: 271–276

Kéri G, Mezö I, Horváth A, Vadász Z, Balogh A, Idei M, Vantus T, Teplán I, Mak M, Horváth J, Pal K and Csuka O (1993*a*) Novel somatostatin analogs with tyrosine kinase inhibitory and antitumor activity. *Biochem. Biophys. Res. Commun* **191**: 681–687

Kéri G, Mezö I, Horváth A, Vadász Z, Idei M, Vantus T, Balogh A, Bokonyi G, Bajor T, Teplán I, Tamás J, Mak M, Horváth J and Csuka O (1993*b*) Structure-activity relationship studies of novel somatostatin analogs with antitumor activity. *Pept. Res* **6**: 281–288

Kéri G, Échegyi J, Horváth A, Mezö I, Idei M, Vantus T, Balogh A, Vadász Z, Bokonyi G and Ullrich A (1996) A tumor selective somatostatin analog (TT-232) with strong in vitro and in vivo antitumor activity. *Proc Natl Acad Sci USA* **93**: 12513–12518

Kéri G, Échegyi J, Horváth A, Idei M, Teplán I, Csuka O, Tejeda M, Gaál D, Örfi L, Szegedi Z and Szende B (1998) Induction of apoptosis and non-apoptotic programmed cell death by antitumor peptides and peptidomimetics. In *Peptides 1996*, Ramage R and Epton R (eds), pp. 83–86. Mayflower Scientific, UK

Laurent-Crawford AG, Krust B, Muller S, Riviere Y, Rey-Fuille, AM, Bechet JM, Montagnier L and Hovanessian AG (1991) The cytopathic effect of HIV is associated with apoptosis. *Virology* **185**: 829–839

Levitzki A and Gazit A (1995) Tyrosine kinase inhibition: an approach to drug development. *Science* **267**: 1782–1788

Mehlen P, Kretz-Remy C, Preville X and Arrigo AP (1996) Human hsp27, Drosophila hsp27 and human αβ-crystallin expression-mediated increase in glutathion is essential for the protective role of these proteins against TNF α-induced cell death. *EMBO J* **15**: 2695–2706

Morimoto RI, Tissières A and Georgopoulos C (1990) The stress response, function of the proteins and perspectives. In: *Stress Proteins in Biology and Medicine*, Morimoto RI and Georgopoulos C (eds), pp. 1–36. Cold Spring Harbor Academy Press: Cold Spring Harbor.

Mosser DD and Martin LH (1992) Thermotolerance to apoptosis in a human T lymphocyte cell line. *J Cell Physiol* **151**: 561–570

Muller M, Strand S, Hug H, Heinemann EM, Walczak H, Hofmann WJ, Stremmel W, Krammer PH and Galle PR (1997) Drug-induced apoptosis in hepatoma cells is mediated by the CD95 (APO-1/Fas) receptor/ligand system and involves activation of wild-type p53. *J Clin Invest* **99**: 403–413

Ozturk M (1996) Molecular biology of liver cancer. In: *Encyclopedia of Molecular Biology and Molecular Medicine*, Vol. 3, Meyers AR (ed) pp. 480–491. VCH: New York

Patel YC, Greenwood MT, Panetta R, Demchyshyn L, Niznik HB and Srikant CB (1995) The somatostatin receptor family. *Life Sci* **57**: 1249–1265

Pirity M, Nguyen VT, Dubois MF, Bensaude O, Hevér-Szabó, A and Venetianer A (1992) Decreased stress inducibility of the HSP68 protein in a rat hepatoma variant clone. *Eur J Biochem* **210**: 793–800

Pirity M, Hevér-Szabó A and Venetianer (1996) Overexpression of P-glycoprotein in heart- and/or drug-resistant hepatoma variants. *Cytotechnology* **19**: 207–214

Roehm NW, Rodgers GH, Hatfield SM and Glasebrook AL (1991) An improved colorimetric assay for cell proliferation and viability utilizing the tetrazolium salt XTT. *J Immun Methods* **142**: 257–265

Schally AV (1988) Oncological applications of somatostatin analogues. *Cancer Res* **48**: 6977–6985

Scudiero DA, Shoemaker RH, Paull KD, Monks A, Tierney S, Nofziger TH, Currents MJ, Seniff D and Boyd MR (1988) Evaluation of a soluble tetrazolium/formazan assay for cell growth and drug sensitivity using human and tumor cell lines. *Cancer Res* **48**: 4827–4833

Sen S and D'Incalci M (1992) Biochemical events and relevance to cancer chemotherapy. *FEBS Lett* **307**: 122–127

Venetianer A, Bajnóczky K, Gál A and Thompson EB (1978) Isolation and characterization of L-cell variants with altered sensitivity to glucocorticoid. *Somatic Cell Genet* **4**: 513–530

Venetianer A, Pintér Z and Gál A (1980) Examination of glucocorticoid sensitivity and receptor content of hepatoma cell lines. *Cytogenet Cell Genet* **28**: 280–283

Venetianer A and Bösze Z (1983) Expression of differentiated functions in dexamethasone-resistant hepatoma cells. *Differentiation* **2**: 270–278

Venetianer A, Pirity M and Hevér-Szabó A (1994) The function of heat-shock proteins in stress tolerance. *Cell Biol Int* **18**: 605–615

Wyllie AH, Kerr JK and Currie AR (1980) Cell death: the significance of apoptosis. *Int Rev Cytol* **68**: 251–306

© 1999 Cancer Research Campaign
